# Frontotemporal Dementia and Late-Onset Bipolar Disorder: The Many Directions of a Busy Road

**DOI:** 10.3389/fpsyt.2021.768722

**Published:** 2021-12-02

**Authors:** Mari N. Maia da Silva, Fábio Henrique de Gobbi Porto, Pedro Maranhão Gomes Lopes, Catarina Sodré de Castro Prado, Norberto Anízio Ferreira Frota, Candida Helena Lopes Alves, Gilberto Sousa Alves

**Affiliations:** ^1^Geriatric Neuropsychiatry Outpatient Service, Nina Rodrigues Hospital, São Luís, Brazil; ^2^Laboratory of Psychiatric Neuroimaging (LIM-21) and Old Age Research Group (PROTER), Department and Institute of Psychiatry, Faculty of Medicine, University of São Paulo, São Paulo, Brazil; ^3^Psychiatry Residency Program, Federal University of Rio de Janeiro, Rio de Janeiro, Brazil; ^4^Neurology Residency Program, Federal Fluminense University, Niteroi, Brazil; ^5^University of Fortaleza (UNIFOR) School of Medicine, Cognitive and Behavioral Neurology Service, Hospital Geral de Fortaleza, Fortaleza, Brazil; ^6^Post Graduation in Psychiatry and Mental Health, Federal University of Rio de Janeiro, Rio de Janeiro, Brazil

**Keywords:** bipolar disorder, frontotemporal dementia, aging, neurodegeneration, frontal syndrome, neuropsychiatry

## Abstract

It is a common pathway for patients with the behavioral variant of frontotemporal dementia (bvFTD) to be first misdiagnosed with a primary psychiatric disorder, a considerable proportion of them being diagnosed with bipolar disorder (BD). Conversely, not rarely patients presenting in late life with a first episode of mania or atypically severe depression are initially considered to have dementia before the diagnosis of late-onset BD is reached. Beyond some shared features that make these conditions particularly prone to confusion, especially in the elderly, the relationship between bvFTD and BD is far from simple. Patients with BD often have cognitive complaints as part of their psychiatric disorder but are at an increased risk of developing dementia, including FTD. Likewise, apathy and disinhibition, common features of depression and mania, respectively, are among the core features of the bvFTD syndrome, not to mention that depression may coexist with dementia. In this article, we take advantage of the current knowledge on the neurobiology of these two nosologic entities to review their historical and conceptual interplay, highlighting the clinical, genetic and neuroimaging features that may be shared by both disorders or unique to each of them.

## Introduction

Frontotemporal dementia (FTD) is a severe neurodegenerative disorder associated with aging and several behavioral and cognitive symptoms, with an overall prevalence estimated in 15–22/100,000 (1). Three characteristic clinical syndromes in FTD may be described: the behavioral variant of FTD (bvFTD), semantic dementia (SD), and primary progressive aphasia (PPA) (1). bvFTD is characterized by progressive deterioration of behavior and cognition, encompassing at least three of six discriminating features: disinhibition, apathy/inertia, loss of sympathy/empathy, perseverative/compulsive behavior, hyperorality, and dysexecutive neuropsychological profile; additional imaging evidence of neurodegeneration and the presence of functional impairment define “probable bvFTD,” as opposed to “possible bvFTD” (2). Bipolar disorder (BD) is a highly disabling condition characterized by periodic mood changes, euphoria, and disinhibition, usually accompanied by cognitive and functional impairment. The core criterion for the diagnosis of BD

I require a manic episode, whereas a depressive and at least one hypomanic episode is needed for the diagnosis of BD II (3). The estimated prevalence of BD may range in adult life from 2.8 to 6.5 percent (4) and in subjects older than 65 years from 0.1 to 0.5% (5, 6). Episodes of mania or hypomania are deemed frequent and reported in 5 and 7% of young adults, respectively (7), albeit the prevalence in elderly subjects is not fully known.

**Figure 1:**
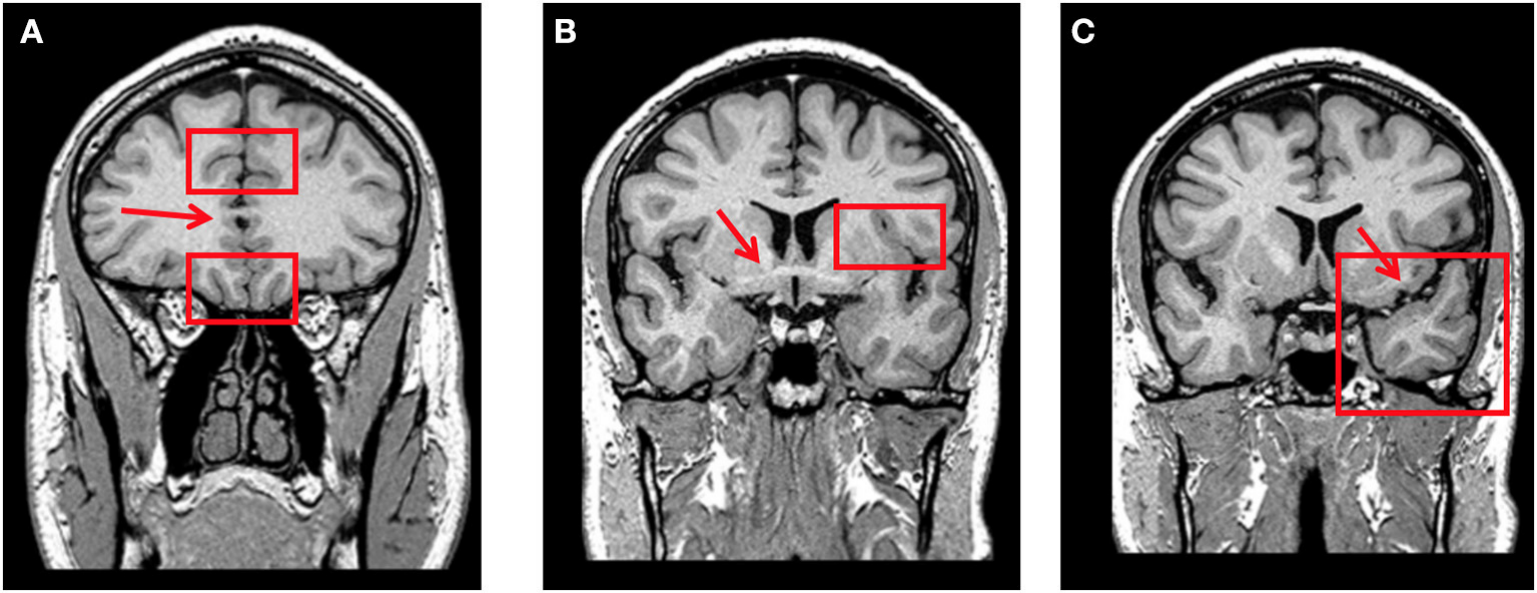
**(A)** Coronal T1-weighted MRI at the level where the corpus callosum is first visible (red arrow). Orbitofrontal cortex is rated through the olfactory sulcus (lower red rectangle) and rostral anterior cingulate cortex through the cingulate sulcus (upper red rectangle) on this slice. **(B)** Coronal T1-weighted MRI at the level where the anterior commissure is first visible (red arrow). Fronto-insular cortex is rated through the circular sulcus (left-side red rectangle) on this slice and the two posterior. **(C)** Coronal T1-weighted MRI at the level where the connection between the frontal and temporal lobes is no more visible (left-side red arrow). Anterior temporal cortex is rated in this slice (left-side red rectangle).

Differential diagnosis of bvFTD with personality or mood disorders is particularly challenging (8). Indeed, whereas standard neuroimaging methods, such as the fluorodeoxyglucose positron emission (FDG-PET), may show a sensitivity of 97% and specificity of 86% for distinguishing FTD from AD (9), the main differential of FTD is with primary psychiatric disorders (10), and some cases will remain unsolved despite neuroimaging and expert evaluation (11, 12) (Figure 1). Many neurodegenerative disorders, particularly FTD, are preceded by affective symptoms, such as mania, racing thoughts, catatonia, and apathy, and numerous genetic of typical FTD symptoms and psychosis have been described (13). One chart review conducted by Wooley et al. found that prior psychiatric diagnosis more often linked to FTD (50.78%) than Alzheimer's disease [(AD) (23.1%)] and subcortical vascular dementia (24.4%) (14). One point is that psychiatric disorders, in general, may be associated with cognitive problems such as working memory, executive functioning, speed of cognitive processing, attention, and episodic memory deficits (15). Brain-age-related abnormalities, particularly volumetric changes in the white and gray matter [(WM), (GM), respectively)], have been increasingly related to the occurrence of late-onset BD (LOBD) and late-onset depression (LOD) (16). This evidence raises the question of whether the presence of a psychiatric illness can be a risk factor associated with an increased risk of dementia in aging. Factors that link BD and FTD include intrinsic and extrinsic factors, such as genetics, number, the severity of manic episodes, interaction with clinical comorbidities, metabolic diseases, long-term alcohol use, cerebrovascular diseases (17–19). Previous neuropsychological studies have linked the number of manic and depressive episodes to an increased cognitive impairment pattern compared with the euthymic state (20, 21). Patterns of neuronal disruption, including frontotemporal-limbic alterations, may be found in both FTD and BD (22). According to the association between BD and dementia (9, 10), Lebert et al. identified a specific type of post-BD dementia with clinical features of FTD (23), and two studies had described a few cases of patients with marked manic symptoms (euphoria, irritability, lack of inhibition, and decreased need for sleep) for the first time in their life and subsequent diagnosis of FTD (14, 24). One FTD study (*n* = 46 subjects) found a positive history for psychiatric diagnosis (BD, schizophrenia, or schizoaffective disorder) in 1/3 of participants with bvFTD (25), although these groups had no differences regarding the familiar history of dementia.

Could severe BD form or, in fact, genuine (neurodegenerative) bvFTD occurring in patients with previous BD, or yet bvFTD that had been mistaken for BD all along? The question is not historical only, for the distinction between BD and bvFTD remains a challenge (10, 11), and the interplay between the two disorders has been increasingly recognized. This narrative review investigates the clinical presentation, genetic and brain imaging changes associated with BD and FTD, particularly bvFTD, analyzing the shared and distinct neuropathological and genetical features and the clinical interchange between both conditions.

## Materials and Methods

A narrative literature review was conducted to gather and summarize the evidence available from original articles for the issue investigated. Our review's guiding question was: “*Which shared and distinct neuropathological and genetical findings may be identified between bvFTD and BD and how these biological markers associate with the clinical presentation in each of the disorders*?” The inclusion criteria were articles in any language since they were available in the electronic databases [(Medical Literature Analysis and Retrieval System Online (MEDLINE), Cochrane, and SCOPUS)]. The paper search included systematic reviews, cross-sectional or prospective design; outpatient or population-based samples of adults over 40 years of age with BD or FTD; the exclusion criteria were guidelines, institutional protocols, papers including other psychiatric or neurological conditions. No specific review interval was set for paper selection.

A search strategy was created to conduct searches in the following databases: MEDLINE via PubMed from the US National Library of Medicine, LILACS, Cochrane, and SCOPUS with no time restriction. To expand our search, we chose to use a natural controlled language. The following descriptors (bold), synonyms, natural language, and Boolean operators were used to cross-check the databases: MEDLINE [Medical Subject Headings (MeSH): search strategy—*(“depression or bipolar depression) and (bipolar or bipolar disorder or mania”) and (“frontotemporal dementia” or frontotemporal lobar degeneration or behavioral variant frontotemporal dementia or behavioral variant frontotemporal or FTD) and (“dementia” or “cognitive dysfunction”)*]. Two investigators independently conducted the literature search and data extraction to minimize selection bias (misinterpretation of results and study design), and any discrepancies were resolved by consensus.

## Historical Notes

Uncommon nowadays, the term “chronic mania” was frequent in the later Nineteenth-century literature (26). While some conceived it as a particular subtype of the illness, with distinctive features since first manifested (27), the prevailing view seemed to be that it represented a late, advanced state of mania (27). Kraepelin called “chronic mania” the state of patients who suffered multiple episodes of mania or had them during aging and did not fully recover (28). Gambogi et al. (25) reviewed his notes on the subject, who observed a striking coincidence between some features of that condition and some of the core features of bvFTD—behavioral disinhibition, loss of empathy apathy (2). Accordingly, in the early twentieth century, Ritterhaus (29) recognized in 1921, amongst the many possible presentations of the manic-depressive illness, one that manifested as “degeneration of the affect,” a description that evokes the modern description of bvFTD (2).

## Differential Diagnosis of bvFTD and BD: Challenges and Boundaries

### The Course and Evolution of bvFTD and BD

Differential diagnosis between bvFTD and BD poses critical therapeutic implications (see case report in Box 1). One is the delay of appropriate medication therapy for manic and psychotic symptoms. Misdiagnosis, conceived by the term FTD phenocopy—introduced by Chris Kipps and John Hodges (30), often leads to clinical dilemma (8) and is treated in the upcoming sections of this paper. One groundbreaking change in the clinical approach of these conditions, both from the neurological and psychiatric perspective, has appeared only recently (13). Indeed, bvFTD and BD should not be viewed as mutually exclusive disorders (13). One must also consider that long-lasting psychiatric disorders, with 2–3 decades of formal diagnosis, may evolve to comorbid bvFTD (31, 32). Late-onset presentation of obsessive-compulsive disorder, psychotic depression, and BD may thus represent the emergence of neurodegenerative disease. In such conditions, persistent psychiatric symptoms may be labeled a “treatment-resistant condition” or “refractory case.” Recent literature has highlighted this limitation, addressing the need for closer collaboration between psychiatrists and neurologists, toward the early recognition of familial mutations of bvFTD, through neuroimaging and genetic investigation (13).

### Psychiatric Features in FTD

#### Depressive Symptoms

BD and bvFTD may both present depressive features, particularly lack of motivation, interest and energy, anhedonia, and impaired concentration (33). However, the specificity and impact of depressive symptoms in each disorder is a matter of controversy. Guilty ruminations, feeling of worthlessness, and suicidal thoughts, although not universally found in BD depression, tend to be even rarer in bvFTD; mood changes and concerns about anhedonia, alexithymia, when present, are reported superficially by bvFTD subjects (33, 34). Accordingly, Woolley and colleagues (35) argue that bvFTD patients are apathetic and emotionally withdrawn, but usually not depressed, for they often lack sadness. This emphasizes the need to carefully investigate the subjective experience, which opposes BD depression to bvFTD. More illustrative of such difference are, however, blunted affect and loss of empathy. Indeed, other authors have reported a high frequency of depressive symptoms in bvFTD patients (36–38). Blass and Rabins have identified three mood syndromes in patients with bvFTD, namely major depression, mood lability, and profound apathy (36). Likewise, in their meta-analysis, Chakrabarty et al. concluded that depressive symptoms were commonly detected in bvFTD patients; however, the authors highlighted the significant heterogeneity of diagnostic methods across studies and the overlap of symptoms of depression with those of bvFTD (37). A recent systematic review showed that depression was highly prevalent in FTD, with similar if not greater frequency in FTD patients than in patients with atypical early onset-DA (38). Nevertheless, the authors also highlight the inconsistency of tools used to assess these symptoms. Of note, in that review, the prevalence of apathy in bvFTD patients was found to be 73–100%.

The sum of evidence supports thus an integrative work up including structured clinical story, social cognitive batteries, and structural and molecular neuroimaging to clarify the nature of depressive symptoms in both bvFTD and BD (10, 33, 39).

#### Manic Symptoms

Manic symptoms may be the first manifestation of bvFTD (40), and BD is one of the most common psychiatric diagnoses to be erroneously given to a patient with bvFTD (10). Symptoms of mania that may be present in FTD patients include irritability, pressured speech, flight of ideas, impulsivity, decreased need for sleep, and psychomotor agitation (35, 41). Besides, they often present with severe disinhibition and may show repetitive motor behavior and stereotypies (2, 42, 43), the latter not commonly presented in mania (44). For most clinicians, the distinction between FTD-related disinhibition and euphoric manic features may be challenging, and a thorough revision of psychopathology is required. One first consideration is the concept of mood liability, usually encompassing cyclothymia or the shift from happiness, expansiveness, or joy to irritation and exaltation (3). The sense of grandiosity and invulnerability, often accompanied by increased energy and decreased need for sleep, is much less reported in FTD than in BD (10). Excessive jocularity and inappropriate social and sexual behavior may occur in both FTD and BD (45), and such findings may be mistaken by the expansive mood that characterizes mania. However, disinhibition in bvFTD is a more pervasive disorder, which may encompass loss of knowledge of social norms, impulsivity, lack of persistence, and motor restlessness (46).

Moreover, as Mendez (41) pointed out, even patients with bvFTD described as having manic symptoms frequently lack truly elevated mood with inflated self-esteem. Instead, some FTD individuals may show a lack of interest in sex (47) and stereotyped and inadequate sexual behavior, such as public masturbation (14), in a more pronounced way than BD. Furthermore, other authors have reported carelessly insulting observations, minor theft, worse financial decisions, lack of remorse, as often reported in FTD (14). Finally, the distinctive nature of disinhibition in bvFTD is reflected in the evidence that this symptom may be improved by antidepressants, such as selective serotonin reuptake inhibitors (SSRI) (48), which contrasts with the expected effect of antidepressants in mania. Finally, the use of mood stabilizers in FTD lacks quality evidence, as the significant side effects must be weighed against its potential benefits to the patient (49).

Box 1 **Case report.**

The patient R.L., a previously healthy young woman, started displaying personality changes by 37 years old. At first, she noticed an increase in her alcohol consumption and smoking habit. She also started to display mood alterations, affective lability, and episodes of impulsivity. During her evaluation by a psychiatrist, she scored 19/39 in the Beck Depression inventory-Short Form (BDI-SF) and 2/13 in the Question 1# of the Mood Disorder Questionnaire (MDQ). She has been prescribed Escitalopram 15 mg in the morning, Olanzapine 2.5 mg once a day, and Alprazolam 0.25 mg by night.

However, during the following months, the clinical scenario progressed with increasing disorganized and repetitive behavior, functional compromise, hyperphagia, and hyperorality. The patient was then committed for 70 days in a psychiatric facility, where the diagnosis of schizoaffective disorder was first proposed. At discharge, the patient took Ziprasidone 80 mg, Clozapine 200 mg, Bupropion 150 mg, Quetiapine 200 mg, and Olanzapine 10 mg per day.

Shortly after her discharge, 1 year after the symptom onset, the patient was taken to our care. Her family stated that she was becoming progressively more apathetic, socially withdrawn, dysexecutive, irritable, and presented alterations regarding her eating habits and speech. Her parents also noticed a decline in her mnestic abilities and attention. The patient behaved in a childlike manner and displayed a lack of self-care. During the physical examination, she presented with dysdiadochokinesia, alterations of tandem gait, and rigidity in both arms. Ziprasidone, Clozapine, and Olanzapine were removed, and her prescription was changed to Quetiapine 100 mg/per day and Bupropion 150 mg/per day. After this drug adjustment, her motor performance improved greatly. Her disorganized behavior and neurological alterations made us think about the possibility of a neurodegenerative disorder, which led us to order a complementary workup.

**Figure 2:**
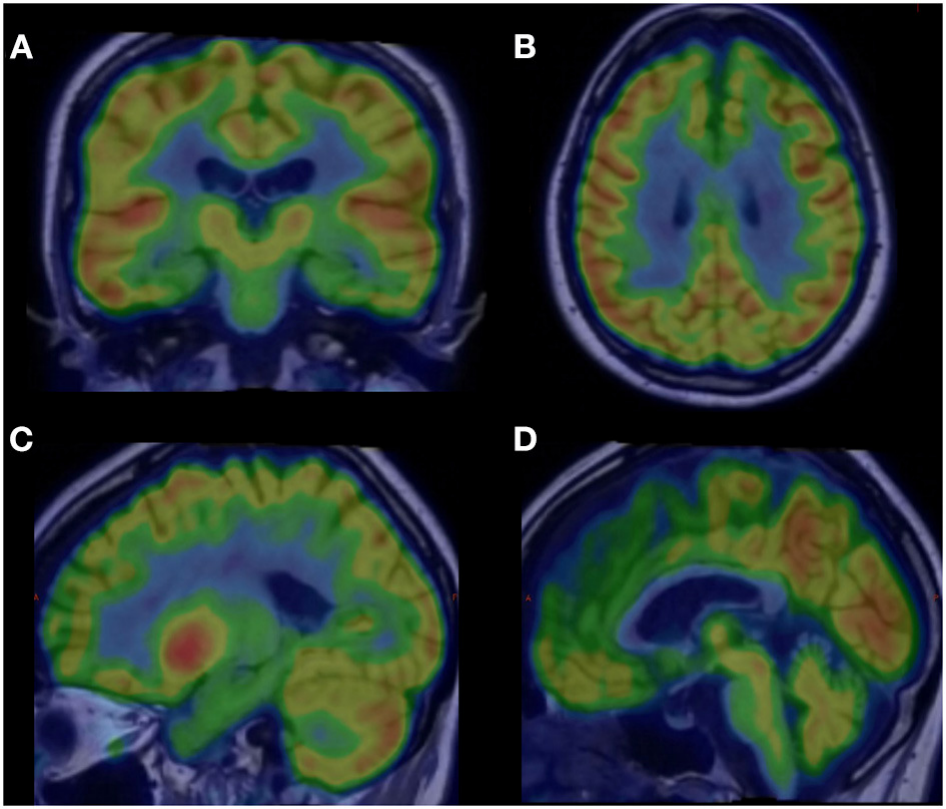
18F-FDG PET/CT slices exhibiting hypometabolism areas (green colored) in FTD: **(A)** hippocampus and mesial temporal lobes bilaterally (coronal slice); **(B)** medial frontal cortex/parafalcine region (axial slice); **(C)** precuneus and posterior cingulate cortex and **(D)** cerebellar vermis (sagittal slices).

She was then submitted to a 18F-FDG PET/CT, which evinced a heterogeneous radio-markable glucose distribution, characterized by a hypometabolism in the medial frontal (parafalcine) and mesial temporal and precuneus regions, limbic system, and superior portion of the cerebellar hemispheres and vermis (Figure 1). The patient also underwent a Magnetic Resonance (MRI), which no expansive, vascular or demyelinating lesion in the parenchyma, MRI spectroscopy exhibited neuronal reduction and an increase in the glutamine and glutamate levels in the frontal lobe and in the anterior margin of the cingulate gyrus (Figure 2). A lumbar puncture was performed, and the cerebrospinal fluid analysis revealed no alterations in cellularity, glycorrhachia, or tau-proteins; VDRL, neoplastic cell research, fungi research, Gram, and BAAR were negative; however, there was a slight elevation of proteinorrhachia (62 mg/dl) and amyloid-beta protein (67 g pg/ml); while neurofilament light chain (NFL) was significantly increased (1,314 pg/m1). Elevation of the CSF NFL may be correlated with the likelihood of a FTD diagnosis (1, 2); this laboratory marker associated with highly suggestive brain imaging and clinical picture allow us to classify this case as a Probable bvFTD with a rapidly progressive presentation in a young patient (3).

**Discussion**

This case provides an interesting discussion regarding the neuropsychiatric differential diagnosis of FTD, especially for its atypical presentations. Without any known comorbidity, a young woman experiencing a new onset of psychiatric symptoms is usually not classified as a very likely case of FTD. Henceforth, one can argue that in this particular ease, the central hypothesis, at first glance, would be of late-onset of a primary psychiatric disorder, such as Schizoaffective disorder, Bipolar Disorder, Schizophrenia, or Psychotic Major Depression. The treatment was subsequently established, but no clinical improvement could be perceived even after several months of high-dose pharmacotherapy. As the patient proceeds to display progressively disorganized behavior, with remarkable compromise of executive functions, volition, mood, personality, memory, and many other mental domains, it becomes clearer that her clinical presentation may not be due to a primary mood or psychotic disorder. The lack of comorbidities and focal neurological signs associated with a progressive and chronic installation supported a neurocognitive disorder.

Concerning Rascovsky's criteria for bvFTD (4) five out of six classic clinical features could be evidenced, such as dysexecutive symptoms, hyperorality, apathy, perseverative behavior, and disinhibition. No other neurological or psychiatric diagnosis seems to explain the patient's clinical picture more satisfyingly. After extensive evaluation, complementary workup evinced alterations of CSF proteins and hypometabolism of multiple cortical and subcortical structures. These findings provide further support to the FTD diagnosis. There are no CSF alterations specific to FTD; however, recent studies indicate a correlation between an elevation of the CSF NIL and FTD diagnosis (5). The functional brain-imaging findings also suggest FTD, even though the limbic and cerebellar hypometabolism are not typical alterations. Nonetheless, it could be theorized that subcortical and cerebellar alterations found in this case may be associated with cortico-subcortical-cerebellar diaschisis, reflecting a broad compromise of multiple behavioral circuitry secondary to frontotemporal cortical damage.

### Cognitive Alterations in FTD and BD

The neuropsychological profile of FTD characteristically involves marked deficits in executive function, verbal memory, and emotional processing (2). Similar deficits are common in patients with BD, especially in the elderly (20, 21). Indeed, cognitive deficits involving attention, executive function, and memory have long been recognized in BD patients during depressive or manic episodes (50); the persistence of cognitive symptoms may be an endophenotype, at least in a subset of BD patients (51). Moreover, early visual-spatial changes (51) and decreased performance in working memory, visual-spatial skills, and inhibitory control may be present even in young and stabilized BD patients (52), whereas significant cognitive deficits may persist after mood episode remission (53). Several cognitive symptoms, particularly affecting executive function, attention, working memory, language processing, and episodic memory, have been reported in LOBD (51, 54).

Not rarely, especially in the elderly, the cognitive impairment is severe enough to fulfill the criteria for dementia (23). Whether this represents a cognitive stage of BD (55), the influence of acute depression or mania, or the interaction of ongoing neurodegeneration, has relevant implications. The occurrence of residual cognitive decline in euthymic LOBD also poses important therapeutic questions. One is the indication of anticholinesterases (AChes), which has been associated with manic-switch in a few reports for dementia (56). Risk factors putatively associated with BD include the number or duration of manic or depressive episodes (57, 58), age of onset (16), use of lithium (59, 60), and cerebrovascular disease (61, 62). Early presentation of bvFTD may include loss of empathy and emotional detachment, leading to misdiagnosis with BD, obsessive-compulsive disorder, or schizophrenia; however, it tends to display less severe cognitive deficits than active primary psychiatric disorders, as demonstrated by Vijberberg et al. (15, 39).

Furthermore, higher scores in two validated scales–Stereotypy Rating Inventory and Montgomery-Asberg Depression rating scale—have distinguished FTD from psychiatric disorders (15). Other distinctive features of bvFTD and BD encompass tests addressing the theory of mind-related domains. Social Interpersonal abilities were investigated in FTD and unipolar depression by Bertoux et al. (63) using Social Cognition Emotion Assessment. Lower scores in a subset of SCEA, including facial recognition skills, inhibitory control, decision making, and apathy, were significantly lower in the FTD group (63).

In summary, neuropsychological tests addressing social cognition, theory of mind, and executive dysfunction may show higher accuracy in differentiating between BD and bvFTD and maybe thus preferred in the clinical setting (63) for early diagnosis and treatment (15, 39, 64).

### The bvFTD Phenocopy Syndrome and Late-Stage BD

Evidence has accumulated, particularly over the last decade, that some patients may present in mid or late life with cognitive and behavioral changes that are identical to that observed in bvFTD patients, albeit they will not progress or indeed show any neuropathological evidence of degeneration; such a presentation has been called “FTD phenocopy” (65). From an operational viewpoint, Devenney et al. (66) defined FTD phenocopy as a syndrome initially meeting criteria for possible FTD, which remained stable after 3 years; patients meet three out of the six core criteria for FTD, exhibit normal brain structural imaging; finally, at a 3-year interval assessment, do not show deterioration in either cognitive tests, daily living activities or brain imaging.

The etiology of the bvFTD phenocopy syndrome has been a matter of great interest (10, 30, 33, 55, 65). Intriguingly, a few patients coming to autopsy did not have any frontotemporal lobar degeneration (FTLD) pathology (12, 66). FTLD-ubiquitin pathology was observed in the *post-mortem* examination of a patient with a very slowly progressive form of FTD, whose son had a similar history in addition to brain imaging that was stable for over a decade (67). However, there is no agreement on whether such patients could be called a phenocopy or a variant of FTD. Remarkably, the C9orf72 expansion, which has been linked to slowly progressive bvFTD (68, 69)—although it has also been reported in some aggressive presentations (70), has not proven to be overrepresented among bvFTD phenocopy patients (66, 70). In addition, the observation that some cognitive deficits, such as poor inhibitory control and emotional processing, are common to both bvFTD phenocopy patients (71) and patients with autism-spectrum-disorder (72), have led to the hypothesis that the former may represent a late-onset, decompensated presentation of the latter (66).

Nonetheless, a case-control study instead disclosed a more significant proportion of personality traits or disorders (avoidant, dependent, or obsessive-compulsive) and a higher rate of adverse life events and relationship problems among bvFTD phenocopy patients as compared to patients with probable bvFTD (73). Furthermore, a progressive aspect of BD, including an end-stage characterized by a cognitive and behavioral change that may correspond to a bvFTD phenocopy, has been increasingly recognized (55, 73). Of note, some studies on bvFTD phenocopy excluded patients with a history of psychiatric disorders (66), preventing a conclusion on a possible interaction. Finally, the role of external biases in the definition of FTD phenocopy has been the subject of discussion mainly when the diagnosis is biased by the proxy, by overestimating cognitive and social behavior impairment, despite the disorder's absence of clear neuro progression.

### The Frontal Syndrome of Catatonia

Not rarely, depressed patients referred to behavioral neurology/neuropsychiatry by general psychiatrists or geriatricians because their cognitive and behavioral symptoms warned investigation of dementia, do have catatonic features. These patients often have longstanding or undiagnosed BD. Most of these features also overlap with or may closely resemble those of bvFTD (2, 74). Indeed, BvFTD itself may manifest as catatonia (74, 75), and the finding of mood-related catatonic states may in some cases also evolve to FTD (76, 77); conversely, it is in the context of its differential diagnosis with primary psychiatric disorders, particularly BD, that the recognition of catatonia is particularly relevant.

Kahlbaum historically introduced catatonia in 1874 (78), a psychomotor syndrome associated with frontal lobe dysfunction (77, 79, 80), may be seen in psychiatric as well as in neurological and general medical disorders (81, 82). In the first group, it is less often a presentation of schizophrenia than mood disorders, being particularly evocative of BD. Both depression and mania may present with catatonic features (79). Features of catatonia, according to the DSM-5 (3), are defined by symptoms comprising stupor, catalepsy, waxy flexibility, mutism, negativism, posturing, mannerism, stereotypy, agitation, not caused by external stimuli, grimacing, echolalia, and echopraxia. The Bush-Francis catatonia rating scale (83), a standardized tool for detecting catatonia, offers a more detailed characterization of the syndrome, including additional rigidity, *gegenhalten*, perseveration, and primitive reflexes. Patients with catatonia due to psychiatric disorders, though, usually do not meet criteria for bvFTD because they lack core features, e.g., insidious onset, gradual progression, and early decline in social behavior (77); not surprisingly though, they may sometimes be hardly distinguishable from patients with bvFTD (10, 77, 84). The diagnostic may be particularly challenging when catatonia shows symptoms that seem specific to bvFTD, such as a craving for sweet foods (84, 85), or when cognitive deficits precede the catatonic presentation.

The clinical similarities between catatonia and bvFTD likely lie on shared neuroanatomic substrates, critically involving the frontal circuitry. Due to primary psychiatric disorders, frontal circuits dysfunctional in catatonia include the prefrontal cortex, the basal ganglia and the thalamus (81), and the orbitofrontal cortex (80). In catatonic patients, these areas and the posterior parietal cortex are thought to harbor very abnormal neurotransmitter activity (83). In particular, as it is reflected on by its singular response to benzodiazepines (86), catatonia is associated with low gamma-aminobutyric acid (GABA) activity in the frontal cortex (87). GABA hypofunction would result from glutamatergic hyperactivity due to the excessive activation of N-methyl-D-aspartate receptor (NMDA-R) (88); in one study, the enhanced response to NMDA-R medications has corroborated such a hypothesis compared to refractiveness to lorazepam among subjects with catatonia (77). Remarkably, GABA and glutamate are both reduced in the prefrontal cortex of patients with bvFTD, in whom these deficits correlate with disinhibition (89). Other neurotransmitters are likely involved in catatonia. The role of dopamine is complex, as illustrated by the observation that atypical antipsychotics may paradoxically worsen and treat catatonia and may consist of dysregulation in the thalamocortical loops; these are strongly modulated by dopamine, GABA, and serotonin projections from the dorsal raphe (90, 91). Electroconvulsive therapy (ECT), the treatment of choice in refractory cases or when benzodiazepines fail (92), increases regional cerebral blood flow in frontal and parietal lobes of catatonic patients (93) and also acts via the GABA system (94). Finally, studies have shown the frequent occurrence of delirium, including hyperactive presentation, among patients with catatonia, particularly during hospitalization (95). Clinical suspicion of comorbid delirium is regarded as a robust predictor of a more thorough general medical workup (neurological conditions), including EEG and neuroimaging (95). Table 1 summarizes the neurotransmitter deficits in FTD according to the literature (89, 96, 97).

**Table 1:** Neurotransmitter deficits in frontotemporal dementia.

**Neurotransmitter**	**Region(s)**	**Findings**
GABA	Frontal, temporal	Reduced
Glutamate	Frontal, temporal, basal ganglia, thalamus	Reduced
Dopamine	Putamen, caudate, substantia nigra, frontal lobes	Reduced
Noradrenaline	–	Likely not affected
Serotonin	Frontal, temporal, midbrain, hypothalamus	Reduced
Acetylcholine	Nucleus basalis	Mildly reduced

*GABA, gamma-aminobutyric acid*.

## Neuroimaging Evidence and Differential Diagnosis of FTD and BD

### Structural Imaging Methods

Structural and functional neuroimaging are integral parts of the investigation in suspected cases of bvFTD and are a powerful tool to differentiate this condition from BD, especially LOBD (10, 33). The presence of atrophy and/or hypometabolism in frontal and anterior temporal lobes increases the likelihood of degenerative over psychiatric disease. Even with clinical criteria of bvFTD fulfilled, the probability of the diagnosis increases from “possible” to “probable” when neuroimaging is altered incompatible brain regions (2). In general, MRI is the preferred method of structural imaging. CT is used if there is some contraindication to MRI. Along with phenomenological heterogeneity, the multiplicity of radiological patterns of both BD and FTD challenges clinical diagnosis, mainly when primary psychiatric disorders are enlisted in the differential diagnosis.

Both bvFTD and BD may be associated with brain atrophy. There are some commonalities between bvFTD and BD, with both disorders causing cortical volumes loss in prefrontal and anterior temporal regions involved in complex behaviors such as the orbitofrontal, dorsolateral, dorsomedial, anterior insula, and anterior temporal, respectively. Cortical atrophy, though, has a more significant magnitude and usually can be seen clinically in bvFTD. Instead, in BD and other psychiatric disorders, cortical atrophy also occurs, but the magnitude is lower and maybe only seen in group analysis with statistical methods (98). Thus, careful examination of MRI through visual rating scales may help characterize subtle atrophy in crucial brain regions typically affected in bvFTD, such as the anterior cingulate cortex, orbitofrontal cortex, anterior insular cortex, and anterior temporal cortex (Figure 1) (99). The presence of atrophy increases the likelihood of bvFTD over BD. Despite that, some cases of early bvFTD may not have cortical atrophy (particularly in genetic cases) or have atrophy outside the frontal and temporal lobe (parietal atrophy) (39).

Noteworthy, the patterns of neurodegenerations in bvFTD are often heterogeneous, and structural and metabolic abnormalities vary considerably (100). Distinctive patterns may be dominantly frontal or frontotemporal, with atrophy broadly restricting these regions, despite the possibility of mild parietal findings. Another pattern is temporal frontoparietal, where medial temporal lobes and parietal lobes are involved, with a lesser intensity of frontal lobe damage than in frontal dominant and frontotemporal subtypes. Finally, the predominant temporal subtype may occur, with the relative sparing of frontal lobes. One specific pattern of bvFTD relevant in this discussion is the right-dominant anterior temporal atrophy where psychotic-like speech (pressure of speech, tangentiality, derailment, clanging/rhyming, and punning) may be present, mimicking a manic phase of BD (41).

Since illness trajectories in BD are significantly variable, neuroprogression is not a general rule in neuroimaging studies (101). Dols et al. (55) reported on five euthymic BD patients with loss of insight and empathy, executive dysfunction, and frontoparietal atrophy; after 3 years of follow-up, brain volume reductions suggesting neurodegeneration were not noticeable (55). In addition, age-related microstructural abnormalities in the WM of elderly subjects were less associated with the onset of BD in the second decade, compared to the outbreak in the fourth and fifth decades (16).

### Molecular and Functional Imaging

Nuclear imaging methods, particularly single-photon emission tomography (SPECT) and 18-fluorodeoxyglucose-positron emission tomography (FDG-PET) are employed to enhance the accuracy in the differential diagnosis of bvFTD and BD (10, 33). FDG-PET is preferred over SPECT because studies showed clear superiority of the former. FDG-PET can help diagnose half of the cases undetermined with structural MRI (102). One cohort study conducted by Vijverberg et al. reported an increase of sensitivity from 27 to 82% when adding neuroimaging findings for the FTDC criteria for possible bvFTD (39). However, there is no perfect accuracy, with some cases showing false-positive results (frontal and anterior temporal hypometabolism in other neurologic disorders or BD) and false-negative results (definitive genetic cases showing normal metabolism) (39). Non-specific or mild hypometabolism, especially in the dorsomedial prefrontal cortex, should be carefully evaluated because it may also occur in psychiatric disorders like BD. Thus, systematic psychiatric follow-up reevaluation of suspected bvFTD—in a minimal period of 2 years–is required in most cases to ensure and validate the baseline molecular findings (39).

Some strategies may help in challenging cases, such as using standardized computer-assisted approaches with quantitative analysis and repeating the exam after 1 year to compare progression (10). In the study of Zhutovsky et al. support vector machine was employed to establish the diagnostic accuracy of clinical and voxel-wise MRI findings over a 2-year follow-up. The findings yielded an average accuracy of 79% (75% sensibility; 86% specificity) in distinguishing FTD from psychiatric disorders (103).

### A Sum of Anatomic-Clinical Evidence in FTD and BD

Cognitive deficits in FTD and BD may be related to several structural brain abnormalities, such as the cingulate cortex and the atrophy of frontal and temporal lobes, which seem to predispose to frontal and temporal circuit dysfunction (18, 51). In the SPECT, frontal and temporal perfusional alterations correlated with “pseudomanic behavior,” “cognitive,” and “pseudodepressed behavior” endophenotypes among FTD (14, 104). Elderly patients with a long time of recurrent depression may present frontotemporal atrophy (105). Decreased metabolism in the superior temporal gyrus was found among elderly BD (106, 107) and bvFTD subjects compared to healthy controls (22). Delvecchio et al. also reported decreased GM volumes in the anterior cingulate cortex in both groups (22); microstructural and metabolic deficits in the superior temporal cortex may be related to the recognition of sounds, and speech processing (Wernicke's area) and the finding of linguistic deficits may be a typical pattern of BD and FTD (108, 109). However, the proportion and localizations of structural and metabolic changes may differ between the two groups. For instance, one study found more significant reductions in BD's ventral lateral prefrontal cortex (PFC), while broader atrophy in dorsolateral PFC and orbital frontal cortex was noted among FTD (22). In the same study, more extended reductions in parietal and limbic lobes and unique volumetric decrease of the posterior cingulate among bvFTD were regarded as a distinctive endophenotype correlated with more severe cognitive impairment in this group (22). Conversely, prefrontal atrophy correlated with executive and social cognitive deficits among bvFTD subjects, but not in the elderly BD group, shedding light on the differential diagnostic of atypical cases (110). Finally, a pattern of asymmetric cortical atrophy with parietal involvement was found in two subjects with FTD and premorbid affective disorder; both subjects shared GRN mutations, notably the g.11019 11022delCACT (111). Another case report of a 64-year-old patient with C9ORF72 mutation and long-lasting premorbid BD; frontotemporal, orbitofrontal cortex, insula, and anterior cingulate were the main atrophic areas (112).

Taken together, the findings mentioned above may support a partial overlap in the pattern of circuitry disruption and anatomical changes shared by BD and bvFTD. Putatively, affective disorders may represent an early preclinical symptom of FTD, at least those who were GNR (113) or C9ORF72 (112) mutation carriers. Many aspects, however, remain disputed. The natural course of the disease may bias more evident brain changes reported in the bvFTD, often diagnosed in clinical stages when neuropathology is advanced, and the small sample sizes involved in the studies.

## Genetic Changes in FTD and BD

Many cases of FTD have dominant inheritance (13) and ~25–40% of FTD cases are familial (111, 114). FTD spectrum disorders can be associated with several different genetic alterations; the prominent representatives of such are the C9ORF72 repeat expansion (most common) and MAPT and GRN genes mutations, while less common mutations occur in the TBK1 (fourth most common), VCP, TARDBP, and CHMP2B genes (115, 116). An increasing number of FTD-related genes, such as the FUS, SQSTM1, CHCHD10, OPTN, CCNF, and the TIA1 mutations (accounting for nearly 5% of cases), have been identified (113). Many studies have also identified a pattern of variability in the heritability of FTD throughout its clinical subtypes; for instance, a more frequent family history in bvFTD in comparison to non-behavioral variants (primary progressive aphasia and motor phenotypes) (117, 118). Furthermore, increasing evidence links the C9ORF72 repeat expansion to a manifestation of atypical psychotic symptoms in carriers before bvFTD (70, 119, 120), including “fixed behavioral patterns in daily life" and limited empathy (120); conversely, most studies reported no direct, clear statistical correlation between C9ORF72 expression and a primary psychiatric disorder (121), except for Gosink et al. (120), who described more significant pathological personality traits early in life in positive rather than negative C9ORF72 carriers.

Twins studies estimate an overall heritability of ~70% in BD, suggesting a significant contribution of genetic factors (122, 123). The most frequently reported genes are CACNA1C, DTNA, FOXP1, GNG2, ITPR2, LSAMP, NPAS3, NCOA2, and NTRK3, and appear to be atypically expressed in the dorsolateral prefrontal cortex of BD (124). Genetic polymorphisms, including variants within the genes CACNA1C, ODZ4, and NCAN (125), is related to a polygenic association of many different risk alleles and environmental factors, not only by the effect of a few strong genes. More recent genome-wide association studies have been performed and identified three genes that might be related to BD: ANK3 located on chromosome 10q21.2; CACNA1C on chromosome 12p13; TRANK1 on chromosome 3p22, and DCLK3 on chromosome 3p22 (126).

As clinical and neuroimaging findings, emerging evidence of overlapping genes linking BD and bvFTD has been highlighted (127). An early study by Lebert et al. proposed a specific type of post-BD dementia with clinical features of FTD (23). In the last 10 years, GNR (128), progranulin mutation (127, 128), and chromosome 17 165 (3′UTR+78C/T) (18) have been reported in case series of BD evolving to FTD (111, 127). Two studies have reported a few patients with the first onset of manic presentation, including euphoria, irritability, lack of inhibition, decreased need for sleep, and later their evolution, conversion to FTD (14, 18, 24). Some studies reported a hexanucleotide repeat expansion in a noncoding region of the C9ORF72 gene as the cause of chromosome 9p21-linked amyotrophic lateral sclerosis, FTD (129, 130). Saladin et al. also reported a case of secondary mania, which progressed to FTD and ALS in a patient with C9ORF72 (19). Tau or TDP-43 depositions have been reported in subjects with SZ and BD (131).

Taken together, these studies support the relevance of genetic risk variants for distinct clinical presentations, including the interpolation of FTD and BD. The relation between genetic basis, aging, and environmental variables is another promising field to be explored by upcoming studies.

## Concluding Remarks

The current review provided a concise overview of the historical and conceptual interplay between BD and bvFTD, highlighting the distinct and shared clinical, genetic and neuroimaging features between both conditions. Despite advances in the field, the differential diagnosis's low accuracy of bvFTD and BD considerably impacts treatment and still brings great uncertainty to clinicians. The neuropsychological profile of FTD characteristically involves marked deficits in executive function, and similar deficits are common in patients with BD, especially in the elderly. Conversely, BD, particularly in its late-onset presentation and in the late stage of its longstanding typical form, shares many features with bvFTD, notably a cognitive dysexecutive profile and emotional blunting. At the extreme, as illustrated by the occurrence of the bvFTD phenocopy syndrome, they may be virtually indistinguishable if not for their different family history and heterogeneous progression. The puzzling picture may also include BD patients developing bvFTD and patients with bvFTD exhibiting psychiatric symptoms resembling mania and depression. In addition, catatonia is a syndrome associated with frontal lobe dysfunction whose features largely overlap bvFTD, more often seen in BD than any other psychiatric disorder. While, thus far, genetic studies have attempted to uncover the multidirectional associations linking genetic predisposition and environmental variables, imaging studies have increasingly supported shared anatomic substrates, critically involving the frontal and temporal circuitry. Notwithstanding all achievements, current clinical-neuroimaging methods still require a clinical follow-up period or, in some cases, *post-mortem* neuropathology to ascertain FTD, making targeted-therapeutic treatment an outlying hypothesis. Multimodal studies have, thus, still a long way to integrate genetic testing, prior personality characteristics traits, neuroimaging endophenotypes, and neuropsychological characterization, which may help establish reliable markers for early diagnosis and therapeutics and better comprehend the interplay between BD and bvFTD.

## Author Contributions

MM: method design, systematic review of literature and results compilation (including creation of figures and tables), writing of the manuscript (abstract, introduction, methods, results, discussion, and conclusions), and selection and organization of bibliographic references. FP: discussion of the theory and method, writing of the manuscript (including case report), creation of figures and text, and critical review. PL: discussion of the theory, writing of the manuscript, creation of figures, and text editing. CS: discussion of the theory and method, writing of the manuscript, critical review, and text editing. NF: discussion of theory, writing of the manuscript, and critical review. CA: discussion of theory and critical review. GA: method design, systematic review of literature, figure editing, writing of the manuscript (abstract, introduction, methods, results, discussion, and conclusions), and selection and organization of bibliographic references. All authors contributed to the article and approved the submitted version.

## Conflict of Interest

The authors declare that the research was conducted in the absence of any commercial or financial relationships that could be construed as a potential conflict of interest.

## Publisher's Note

All claims expressed in this article are solely those of the authors and do not necessarily represent those of their affiliated organizations, or those of the publisher, the editors and the reviewers. Any product that may be evaluated in this article, or claim that may be made by its manufacturer, is not guaranteed or endorsed by the publisher.

## Abbreviations

AD: Alzheimer's disease
AChes: anticholinesterases
BD: bipolar disorder
bvFTD: behavioral variant of frontotemporal dementia
CSF: cerebrospinal fluid
CT: computed tomography
C9ORF72: C9ORF72 repeat expansion
FDG-PET: fluorodeoxyglucose positron emission tomography
FTD: frontotemporal dementia
GM: gray matter
LOBD: late-onset bipolar disorder
LOD: late-onset depression
MRI: magnetic resonance imaging
NfL: neurofilament light chain
PFC: prefrontal cortex
PPA: primary progressive aphasia
SD: semantic dementia
SPECT: single photon emission tomography
WM: white matter.
